# DeKalb County Medical Protective Society

**Published:** 1879-08-20

**Authors:** 


### SOCIETY REPORTS.

We give place to the following plan of an organization sent us by a subscriber, adopted by a number of medical gentlemen in a certain locality to protect themselves against the imposition of those who procure medical services for nothing, passing from one physician to another and never paying a bill. The document, we doubt not, will interest many of our readers, and may furnish valuable suggestions in forming like protective societies elsewhere. It is an experiment, having been in operation only about three months. The first effect, that of making everybody mad, is beginning to subside, and the medical friend who sends the paper says: "My collections have largely increased, while my labor has greatly diminished since the adoption of this plan; and as to their getting mad, so far as I am concerned, they may do so and go to the devil, as I practice medicine for a living and not for accommodation." [ED.]

### CONSTITUTION AND BY-LAWS OF THE DEKALB COUNTY MEDICAL PROTECTIVE SOCIETY, DEKALB COUNTY, GEORGIA.

### PREAMBLE.

We, whose names are hereunto annexed, agree to form ourselves into a society, the object of which shall be the advancement of medical science and the adoption of a plan by which the members of this society can more effectually collect fees for medical services.

### CONSTITUTION.

ART. I. *Name.*—This society shall be known by the name of The DeKalb Medical Protective Society.

ART. II. *Officers.*—Section 1st. The officers of the Society shall be a chairman, secretary and treasurer, who shall be elected semi-annually, at such times as may be agreed upon by the Society.

Section 2. It shall be the duty of the chairman to preside at each meeting of the Society, to enforce the constitution and by-laws, to appoint all committees, etc.

Section 3. It shall be the duty of the secretary of this Society to keep a book in which shall be recorded the names of all the members of this Society, and it shall be the duty of the secretary to call the roll at the opening of each meeting of the Society, to read the minutes of the previous meeting and to furnish to each member a correct list of all defaulting patrons reported to this Society.

Section 4. It shall be the duty of the treasurer of this Society to take charge of all money belonging to the Society, to keep a fair and impartial account of the same, and to pay out such money when he may be called upon by the Society.

ART. III. *Members.*—Section 1. No person shall become a member of this Society who is not a regular practitioner of medicine and who does not agree to be governed by the constitution and by-laws.

Section 2. Any member of this Society may be expelled by a two-third vote of all the members present, for a violation of the constitution and by-laws.

ART. IV. *Of Amendments.*—Section 1. This constitution or any section of it may be changed or amended by a two-thirds majority of all the members of the Society, provided one month's notice of the proposed change is given at a regular meeting of the Society.

BY-LAWS.

Section 1. This Society shall meet regularly on the first Monday of each month at 9 o'clock a. m.

Section 2. It shall be the duty of each member to attend regularly at each meeting of the Society, and any member failing to attend, and not rendering a good excuse for absence, shall be fined not less than twenty-five nor more than fifty cents, at the discretion of the Society.

Section 3. All daily, weekly or monthly laborers, receiving wages, and all persons in any business bringing in regular returns, will not be indulged longer than sixty days after the service is rendered; all other persons not longer than the first of November of each year. And it shall be the duty of each member to report to this Society at each meeting the names of all persons who fail to comply with the above terms; provided that any member, for just and sufficient reasons, may extend a longer indulgence, satisfactory arrangements being made.

Section 4. It shall be the duty of each member of this Society to keep a list of all defaulters reported to this Society, and any member attending a defaulter in case of sickness or injury shall be fined not less than five dollars, or expelled, unless excused by the Society by reason of the special emergencies of the case.

Section 5. Any member of this Society is at perfect liberty to practice for genuine paupers at any time, free of charge.

Section 6. It shall be the duty of each member of this Society to make the following charges for medical services, unless it be a cash payment, and then they may make a deduction of 50 per cent.

Mileage, per day, 50 cents per mile; night, $1.00 per mile; and $1.00 for prescription or visit; obstetric fee, $10.00 to $25.00.

The undersigned pledge themselves to abide in good faith the conditions of the above constitution.

*Names of Members:* T. T. KEY, M.D., Chairman.
F. P. H. AKERS, M.D., Secretary.
LOUIS A. GUILD, M.D., Treasurer.
A. H. BRANTLY, M.D.
JAMES G. MAUL, M.D.
A. S. MASON, M.D.

